# Does a video clip enhance recruitment into a parenting trial? Learnings from a study within a trial

**DOI:** 10.1186/s13063-020-04779-0

**Published:** 2020-10-15

**Authors:** Holly C. Mattock, Rachael Ryan, Christine O’Farrelly, Daphne Babalis, Paul G. Ramchandani

**Affiliations:** 1 Centre for Psychiatry, Imperial College London, London, UK; 2 PEDAL Research Centre, Faculty of Education, University of Cambridge, 184 Hills Road, Cambridge, CB2 8PQ UK; 3 Imperial Clinical Trials Unit, Imperial College London, London, UK; 4 Department of Brain Sciences, Faculty of Medicine, Imperial College London, London, UK

**Keywords:** Recruitment, Patient information, Research methodology, Video clip

## Abstract

**Background:**

Reaching recruitment targets in randomised controlled trials is a challenge. Media tools are increasingly used to engage participants, yet there is a paucity of research into the use of video to optimise recruitment. We therefore tested whether adding a participant information video clip to a standard participant information sheet improved recruitment into a parenting trial.

**Methods:**

One hundred seven participants were randomised to receive either a participant information sheet (*n* = 51) or an informational video clip (*n* = 56) as part of an email contact following a screening phase. All participants went on to receive the information sheet as part of the existing consent procedure.

**Results:**

The video condition did not increase the odds of recruitment into the trial, such that those in the video condition were significantly less likely to participate in the main trial (OR = 0.253, CI = 0.104–0.618, *p* = 0.003).

**Conclusion:**

The introduction of a video clip into the recruitment stages of a parenting trial did not lead to an improvement in recruitment; however, the small sample size precludes definitive inferences. We offer reflections on challenges encountered in implementing the SWAT and suggestions for other researchers seeking to embed recruitment SWATs into similar trials.

**Trial registration:**

Current controlled trials ISRCTN 58327365. Registered on 19 March 2015.

**SWAT registration:**

SWAT 106; Effects of a video clip on recruitment into a randomised trial. Registered on 20 December 2016.

## Background

Randomised controlled trials (RCTs) are considered the gold standard in research design for evaluating interventions [1, 2]. However, issues surrounding recruitment and retention are common, which often lead to delays, extra costs, protocol changes or the altogether abandonment of trials [3]. Poor or slow recruitment, whereby the target sample number is not reached within the anticipated timeframe, can lead to smaller sample sizes, which can give rise to sampling bias, limiting statistical power, and may increase the possibility of a type 2 error [4, 5]. For example, within the UK, the National Institute for Health Research (NIHR) invests substantial funding in health intervention research; in 2014 and 2015, this amounted to £237.6 million, with a considerable proportion allocated to RCTs [6]. Yet in Raftery et al.’s [3] review of 125 NIHR-funded RCTs, the authors found that 43.1% of studies submitted a time extension request citing recruitment issues. Walters et al. [6] reported that only 56% of 151 studies conducted under NIHR funding in the UK between 2004 and 2016 reached their recruitment target. Due to the time, cost, and resource implications of recruitment issues, the identification of more effective recruitment methods has become a priority [7, 8].

### Study within a trial

A developing area of trial methodology, which can be used to investigate recruitment strategies, involves embedding a study within a trial (SWAT). SWATs are a means of evaluating methodology and formally assessing how effectively a study is running. Treweek and colleagues [9] defined a SWAT as a ‘self-contained study that has been embedded within a host trial with the aim of evaluating or exploring alternative ways of delivery or organising a particular trial process’ (p. 143). The experimental design of SWATs means they are valuable for testing hypotheses and investigating whether traditional approaches adopted historically can be refined or adapted as research practices evolve. As Shah points out in the paper by Gheorghiade et al. [10], it is ironic that we use trials to generate evidence-based treatments, whereas we rely largely on anecdotal approaches as to what we think works to inform their conduct, rather than developing empirical evidence to identify best practice.

The value of SWATs is increasingly being recognised, with several developments moving to raise their profile and encourage their routine use in RCTs [11]. These include online research platforms specifically designed to address recruitment and retention challenges within RCTs, such as Trials Forge [12] and Clinical triAls [13]. These online resources primarily aim to share knowledge from existing studies and build a platform to collate the findings and inform new hypotheses. Another initiative, the Prioritising Recruitment in Randomised Trials [PRioRiTy; 14] study has identified leading priorities concerning recruitment and retention into RCTs, using a priority setting partnership approach involving the public, carers and healthcare professionals. Amongst the ten leading questions identified were the need to identify how best to design and deliver information on RCTs to the public and the advantages and disadvantages of using technology during recruitment. This focus is timely as the dramatic influx of technology into everyday life provides opportunities to enhance practice using alternative media to present trial information in an efficient, interactive, and potentially low-cost way, for example through video clips, social media and/or informational websites.

Currently sharing an information sheet or form is the conventional format for providing study information to potential participants. Traditionally, this information sheet or participant information sheet (PIS) is a printed text document that aims to provide potential participants with comprehensive details of the study. These often become long and complex to ensure accordance with ethical guidance [15] and may not be visually appealing [16]. Previous research has shown that after receiving a PIS, participants may still not be fully informed, often failing to understand key aspects of the study, such as the right to withdraw [17] and potential side effects [18]. Thus, it is likely that the traditional PIS could be a barrier to study participation for certain demographic groups. In light of these potential limitations of the PIS, the Health Research Authority in the UK has advocated for the investigation of alternative media for participant material [19].

Indeed, there has been an increase in the use of multimedia implemented within trial design (e.g. websites, social media, audio and video clips). It has been suggested that video clips may be an acceptable means of promoting understanding of participation in RCTs with a small but growing number of studies assessing their utility. Meropol et al. [20] looked at the use of a web-based platform to explain key features of a cancer RCT and found that video clips (when compared to text) increased patient knowledge and decreased attitudinal barriers. However, a recent Cochrane review investigating recruitment strategies, which included the effects of video clips versus standard information in three oncology trials, found that there was insufficient evidence to draw conclusions about the effects of clips [21].

More recently, Jolly et al. [22] conducted a recruitment SWAT in a RCT for people with mild symptoms of chronic obstructive pulmonary disease to compare the use of a multimedia resource and standard PIS materials. The multimedia resource shared information about the RCT and included video clips of patients discussing their experiences of participation. No group differences in enrolment were found, although the authors suggested that the multimedia format may not have been best matched to the information preferences of the participants (largely men over 70 years) and may be better suited to populations with higher general consumption of multimedia. Related to this, the TRECA study (Trials Engagement in Children and Adolescents) [23] has conducted user testing to explore the use of a multimedia platform in healthcare trials with children, adolescents and their parents [24]. Participants commented positively about the interactive resource, with varied preferences of information medium; some preferred animations and video clips whereas others preferred text. These resources are due to be embedded and tested in six healthcare trials.

Research using SWATs to investigate the use of multimedia in recruitment processes could identify more effective and efficient recruitment methods in RCTs. As the utility of multimedia may differ depending on the target group, there is a need to investigate its inclusion during recruitment of diverse patient and public populations. SWATs themselves create a vital opportunity to evaluate methodology in real world trials; however, reporting of SWATs remains limited. Through sharing the challenges faced and the key learning points from the present study, we hope to provide information to help support the routine inclusion of SWATs in RCTs.

### Aim

This study aimed to use a SWAT to explore whether the inclusion of a video clip alongside a standard PIS improved the rate of recruitment, compared to a standard PIS alone. We further aimed to report our experience of embedding a SWAT during an active RCT and the challenges encountered, and offer reflections for consideration by other researchers.

## Method

### The host trial—Healthy Start, Happy Start

The present SWAT is hosted by the Healthy Start, Happy Start (HS,HS) study. The HS,HS study is a UK-based RCT funded by the NIHR. HS,HS aims to assess the effectiveness and cost-effectiveness of a Video-feedback Intervention to promote Positive Parenting and Sensitive Discipline (VIPP-SD) in preventing enduring behavioural problems in young children aged 12–36 months (see Ramchandani et al. [25] for further details of the protocol).

#### Eligibility for HS,HS

Participants for HS,HS were recruited predominantly through health visiting services in 6 National Health Service (NHS) trusts, via face to face contact and mailshots. The eligibility criteria for HS,HS included parents aged ≥ 18 years, child aged between approximately 12 and 36 months, a score in the top 20% on population norms for child behaviour problems using the Strengths and Difficulties Questionnaire (SDQ; Goodman [26]) and written informed consent. Exclusion criteria included if the child or parent had a severe sensory impairment, learning disability or language limitation which is sufficient to preclude participation in the trial; were actively participating in family court proceedings; if the parent was participating in a closely related research trial; and/or if the families were concurrently receiving an individual video-feedback intervention.

### Study within a trial rationale

As is the case in many RCTs, recruitment for the HS,HS trial was initially slow, and therefore, an extension was granted for 9 months to enable the target sample size to be met. The HS,HS trial encountered challenges to recruitment in part due to changes to local health visiting services and NHS service provision which impeded the availability of staff time to support face-to-face recruitment. Consequently, the trial increasingly utilised mailshots to reach potential participants. These challenges also stimulated a dialogue surrounding alternative methods to optimise families’ initial engagement in the study and the recruitment process. We had previously revised the PIS to improve its readability and added a randomisation schematic to aid understanding. However, we were constrained by the PIS’s traditional text-based format, ethical requirements concerning the level of detail and topics requiring standardised institutional wording. Thus, we were interested in determining whether adding a short video clip to the PIS could be more appealing, help to personalise the research by introducing the research team and provide sufficient initial information to increase families’ engagement with the recruitment process. Also, as the intervention under study in the HS,HS trial used video clips of parents’ behaviour to enhance parenting, the clip could help to normalise the experience of being on camera. To explore this idea further the team decided to implement a SWAT 8 months into recruitment, after approval was sought through a substantial amendment. The SWAT sought to compare the effect of a video clip and PIS, to the PIS alone, on recruitment and to learn from both participant and researcher feedback, as well as reflecting on the process of embedding a SWAT in an active RCT.

#### Video development

The HS,HS study had an active patient and public involvement (PPI) group that were involved in all aspects of the trial and were also consulted during video clip development. An indicative draft of the video was made by the research team and a dedicated PPI meeting was held to discuss the video. The group’s suggestions shaped the contents and design of the clip, which included adding an explanation of why the study is relevant for the population, highlighting the importance of research within the NHS, timing visual animation effects to match the voice over, what actors should demonstrate in terms of family participation and the inclusion of both senior (e.g. chief investigator) and junior staff (e.g. research assistants completing the research visits) in the video. Following these suggestions, a final version of the video was designed. A member of the PPI group and their family featured in the video to help explain what would be involved in the research visits. The video included a welcome by the chief investigator, outlined what would be involved in each trial arm and introduced the research assistants who would be conducting the home-based research visits. The duration of the video totalled 302 s (5 min).

The development of the video required approximately 60 h of research assistant time. Video props and technological equipment were purchased for £250 and the actor family in the video was given a £50 honorarium for taking part. Both the video producer and graphic designer worked at a significantly reduced rate due to the nature of the research and were each given a £200 honorarium. An alternate quote for video production was £750. Costs for the PPI group consultation were approximately £350. The six attendees were provided with £50 vouchers as compensation for their meeting attendance, email contact and feedback about the design and content of the video, and approximately £50 spent on refreshments at the meeting. The total estimated costs for the development of the video are £1000 (not including researcher time).

#### PIS development

The PIS comprised of a 4-page word document structured in headed paragraphs describing the HS,HS study. The PIS included information on why participants had been asked to take part, the possible benefits and disadvantages of participation, confidentiality, information security, information about the funders and contact details of the principal investigator and trial manager. The PIS was amended during the trial phase of HS,HS to enhance understanding and readability following informal feedback from trial participants. Two schematics were included to illustrate the possible randomisation allocations to improve understanding of group allocation.

#### SWAT eligibility and implementation

**Fig. 1:**
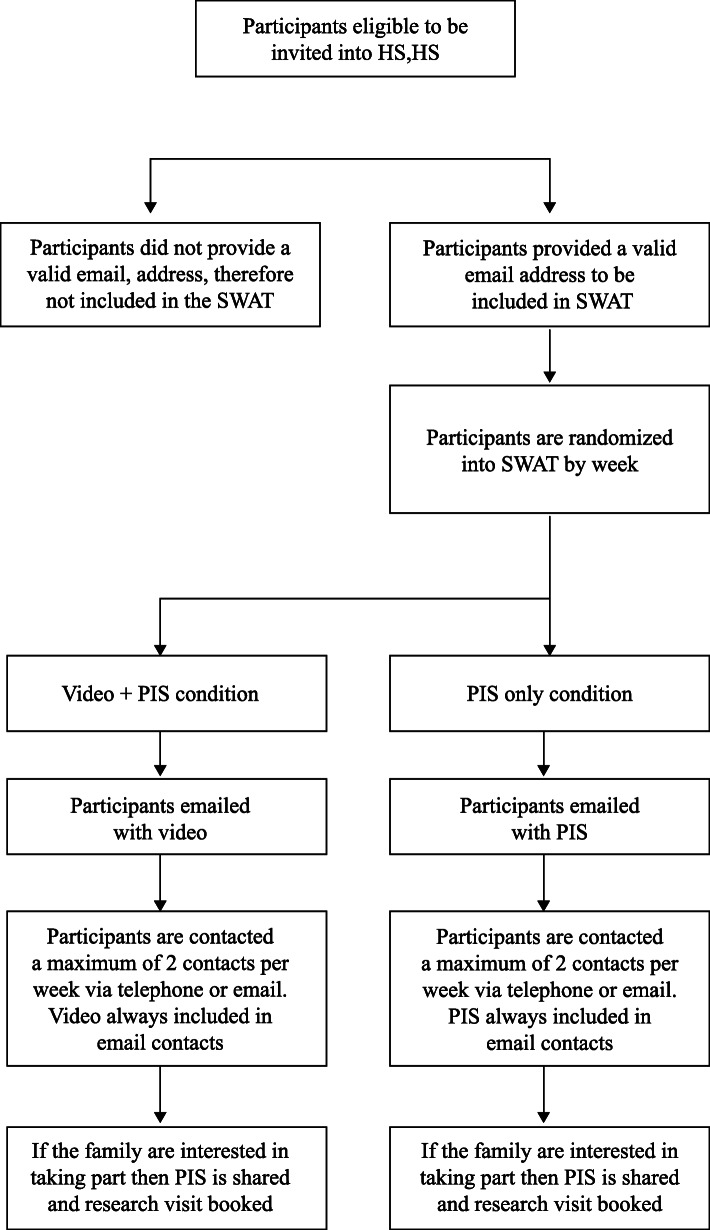
SWAT participant contacts

The SWAT ran for 15 weeks between March and July 2017 until recruitment to the trial ended. Once eligibility for the trial phase of HS,HS was confirmed, participants were assessed for inclusion into the SWAT sample. The additional eligibility criteria required for SWAT inclusion was that participants needed to provide a valid email address. For example, those participants who only provided a telephone number as contact details were excluded. All participants who were invited into the trial during this time period were assessed for eligibility for the SWAT. Participants who were already participating in the HS,HS trial could not be included in the SWAT. See Fig. 1 for information about how participants were contacted.

Participants were randomly allocated to receive one of two initial contacts from the research team: video and PIS or PIS alone. For ease of understanding the video and PIS group will be referred to as the ‘video group’ and the PIS only group will be referred to as the ‘PIS group’ hereafter. A randomisation list for the initial contact was prepared by the trial statistician, and allocation was released weekly to the research team. Each participant was allocated to their condition based on the week they returned their screening questionnaire. The research team were not blinded to group allocation.

The initial randomised contact took place after participants completed the screening phase. Participants were sent an initial email thanking them for completing screening and informing them that a member of the research team would be in touch by phone within a couple of days. The email sent to both groups was identical except that the video group’s message included a link to the video clip, whereas the PIS group only had the trial’s standard PIS attached. Once a minimum of 24 h had passed, the research team called participants to ascertain their interest in participating in the full trial. Follow-up involved telephone call(s) and/or email(s) until contact was made.

The contacts were spread over approximately 4–6 weeks, and there was a mean of 2.7 contacts per participant during this time frame (range 0–15). If contact was established with the participant, the study was explained by a member of the research team and they were then sent an email with the PIS attached, irrespective of group allocation, to ensure this did not affect the provision of the standard PIS to all participants at the time of booking a first research visit.

Participants who were randomised into HS,HS during the SWAT period were then asked a few questions at the end of their first research visit. The aim of these questions was to ascertain feedback about their experience of receiving either the PIS or video clip, whether it affected their decision to take part in the study and how useful they found it (Additional file 1: Appendix 1). Two members of the research team who were responsible for contacting prospective participants during recruitment were also asked about their experience at the end of the SWAT including the ease of implementing the SWAT and their perception of how it influenced participant understanding (Additional file 1: Appendix 2).

#### Analysis strategy

The primary outcome of interest was the proportion of participants who consented to take part in the main trial based upon their SWAT allocation. Therefore, a binary logistic regression was used to examine the main effect of SWAT allocation (video vs PIS) on the number of participants recruited into the trial (randomised vs not randomised). Despite randomisation, more participants in the PIS group (63%) were screened in person in comparison to other methods whereas only 40% of participants in the video clip condition were screened in person. Differences were identified between the groups in regard to baseline characteristics in education and number of contacts made by the team. Therefore, the regression was repeated to adjust for number of contacts made and education status. Educational status was defined in two levels (university and pre-university qualifications) and number of contacts made by the team was continuous. Findings were presented using odds ratios (OR), and 95% confidence intervals (CI) were calculated to compare intervention strategy on recruitment.

A secondary outcome of interest was participant and researcher attitudes towards method of recruitment via brief structured interviews. Our goal was to invite all SWAT participants to participate in an interview; therefore, data saturation was not used to inform data collection. However, due to time demands in research assessments, it was not possible to interview all SWAT participants. Where interview data were available, content analysis was used to analyse the text [27]. However, formal comparisons between the groups were not conducted on frequency of categories, due to the small sample size and differences in group sizes, video (*n* = 5) and PIS (*n* = 12), which increased the risk of bias.

## Results

### Participants

During the SWAT period, 317 potential HS,HS participants were screened, of whom 121 were eligible for the HS,HS trial, and of these, 107 were eligible to be included in the SWAT sample. Fourteen participants were excluded as they had not provided an email address. Of the 107 participants, most were identified as ‘White’ (79%), biological mothers (93%) and educated to postgraduate level (47%). The mean parent age was 33.59 years (*N* = 105, *SD* = 5.4), and the mean child age was 21.9 months (*SD* = 0.5); slightly more male than female children participated (58% vs. 41%). Table 1 displays the participants’ demographic characteristics.

**Table 1:** Demographic characteristics of the SWAT participants

	Video clip *N* (%)	PIS *N* (%)	Interviewed *N (%)*
Mean parent age (years)	33.9	33.3	36.7
Mean child age (months)	22.4	21.4	23.7
Relationship to child			
Biological mother	47.0 (94)	52.0 (91)	14.0 (82)
Biological father	3.0 (6)	5.0 (9)	3.0 (18)
Education level			
Pre-GCSE	0.0 (0)	1.0 (2)	0.0 (0.0)
GCSE	2.0 (4)	1.0 (2)	1.0 (6)
A Level/NVQ/College	11.0 (22)	13.0 (23)	2.0 (12)
Undergraduate	15.0 (40)	12.0 (21)	2.0 (12)
Postgraduate	20.0 (30)	30.0 (53)	12.0 (71)
Missing	2.0 (4)	0.0 (0)	0.0 (0)
Ethnicity			
White	38.0 (76)	47.0 (82)	17.0 (100)
Asian	3.0 (6)	6.0 (11)	0.0 (0)
Mixed/multiple ethnic groups	1.0 (2)	1.0 (2)	0.0 (0)
Black/African/Caribbean	4.0 (8)	2.0 (4)	0.0 (0)
Other ethnic groups	3.0 (6)	1.0 (2)	0.0 (0)
Missing	1.0 (2)	0.0 (0.0)	0.0 (0)
Child gender			
Male	24.0 (48)	38.0 (67)	10.0 (59)
Female	25.0 (50)	19.0 (33)	7.0 (41)
Missing	1.0 (2)	0.0 (0.0)	0.0 (0.0)

*PIS* participant information sheet, *SWAT* study within a trial

**Fig. 2:**
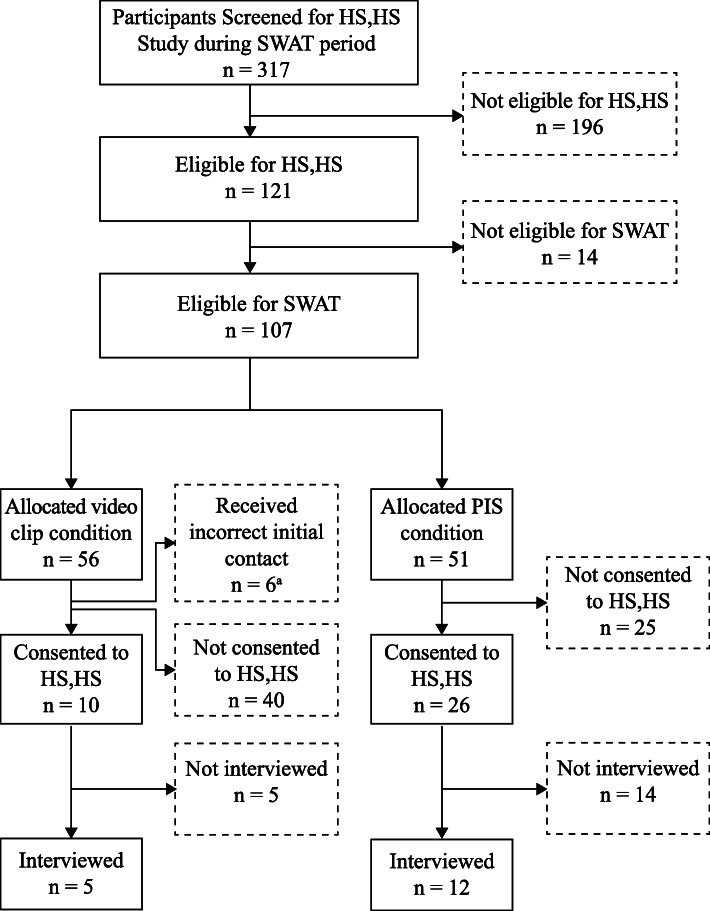
Participant flow through the study within a trial. ^a^Due to researcher error in the context of the busy trial, 6 families were contacted using the incorrect allocation. These participants were excluded from formal analyses

The final sample consisted of 101 participants (50 randomised to video and 51 randomised to PIS condition). There were initially 56 participants allocated to the video condition; however, 6 were then excluded from analysis as they were inadvertently allocated to the wrong group. Figure 2 shows the flow of participants through the study.

### Primary outcome

Of the 50 participants given the video clip, 10 (20%) consented to take part in the trial, compared with 26 (51%) of 51 participants given information only via the traditional PIS. The difference was 26%. Table 2 shows that the odds of consenting to the trial were 76% less in video group than in the traditional PIS group (OR 0.240, 95% CI 0.099–0.582). The OR was not significantly influenced by frequency of contact or by educational attainment (OR 0.253, CI: 0.104–0.618, *p* = 0.003). Thus, the odds of consenting to the trial were statistically lower in the video group compared to the standard PIS group.

**Table 2:** Results of logistic regression analysis for variables predicting randomisation into host trial

	B	SE	Odds Ratio	95% CI	
				Lower	Upper
Step one					
Constant	.039	.280	1.040		
SWAT Allocation	− 1.426	.451	.240**	.099	.582
Step two					
Constant	.297	.399	1.346		
SWAT Allocation	− 1.374	.455	.253**	.104	.618
Education	− .203	.520	.816	.295	2.262
Frequency of contact	− .080	.098	.923	.762	1.119

**p < .01

### Video clip engagement data

The video was emailed to 50 prospective participant email accounts. Access to online metadata from the host website revealed that the link was accessed 38 times, and of these, 16 watched the entirety of the video clip. The average length of play was 190 s (of a possible 302 s).

### Qualitative feedback

Whilst the initial aim was to gain feedback from all SWAT participants who were randomised (*n* = 36), for practical reasons (e.g. time demands of the assessment) and the importance of not overburdening participants, 17 participants were invited to interview. All 17 participants who were approached agreed to interview (5 video, 12 PIS). Baseline demographics of interviewed participants are displayed in Table 1.

Irrespective of condition, all participants commented positively on how useful they found the introductory information. Participants from both groups commented that the information was easy to understand and that it was informative:

“I did find it helpful to watch it and thought that it gave quite a lot of information”. (video clip)

“it was well explained”. (PIS)

However, participants within both groups stated that they had further questions that needed discussing over the phone.

One PIS participant commented: “I think I needed a few things explained”.

And one video clip participant indicated “I think I got more from actually chatting and asking questions that the actual video clip itself”.

The participants in the video group tended to frame the material as introductory, whilst those in the PIS described the material as comprehensive.

The key finding from both researcher interviews was the shared perception that the initial email contact increased participants’ receptivity to the study team and engagement in the trial. Prior to the SWAT being implemented, participants were first contacted by telephone after completing the screening questionnaire. Notably, both researchers reported that participants, regardless of condition, were more open to phone calls after receiving an initial email.

“I noticed a difference in how receptive they were just to our phone call [erm] because they’d had a chance -whatever they’d received they’d had the chance to consider the study a bit more”.

Both researchers found that participants had often not watched the video clip or only viewed a portion of it, although those who had viewed it were reported as having a better understanding of randomisation:

“they all commented on the understanding of the two groups”.

“that was the hardest thing to explain to participants without a visual aid, over the phone I think it was helpful that they had seen those two groups”.

The researchers also highlighted the advantage of participants seeing images of the research team and activities in the video clip:

“I think for the families who had seen it I think that was really reassuring that it’s like ‘look we’re these harmless people who are coming to play with some toys and ask some simple questions’ it’s not this weird abstract visit that has no context”.

## Discussion

Contrary to our expectations, the inclusion of a video during recruitment did not result in an improvement in recruitment rates in this RCT of a parenting intervention. However, the interpretation of the results should be made cautiously given the small sample size and the exploratory nature of the study. This finding is consistent with both the findings of Jolly et al. [22] and Campbell et al. [28]. Campbell et al. [28] simulated recruitment of parents from low-income backgrounds and found no difference in rates of agreement to enrol when comparing various formats of consent including text, narrated video clips and PowerPoint presentations. Participants reported no greater preference for video clip over text, although when accounting for educational attainment, those who read at an 8th grade level or below did show improved comprehension for an enhanced text version of the information sheet. However, interestingly even though the preference was specified, there were no differences seen in rates of enrolment. Thus, it is possible that media such as a video may be beneficial in enhancing understanding of research participation, without that understanding necessarily translating into increased recruitment. Indeed, it is conceivable that enhanced understanding of what research participation involves may lead fewer participants to enrol, if, for example, the demands of participation exceed the time participants may be able to commit to the study.

Moreover, participants included in this SWAT were generally highly educated, particularly in those interviewed, and so have an assumed higher reading level. Therefore, it would be plausible that the enhanced PIS used was accessible and understandable. By extension, the video may not have had as much impact on understanding within this population, compared to a potential participant population who have a lower reading ability or based on previous research [28]. Equally this may have been a matter of preference, which may explain the low number of views of the video. One parent specified they were unable to watch the video without headphones in case of waking their child and would have therefore preferred a PIS.

Another possible explanation for these findings may be linked to the format and use of the video clip. The metadata indicated that only one third watched the clip in full, assuming no participant watched it twice. Although studies on patient engagement with clips do not typically report metadata, research on the use of educational videos in the context of distance learning has found that the rate of in-video dropout is positively correlated with video duration, with a predicted drop-out rate of 53% for a 5-min video [29]. Thus, it may be instructive to examine whether a shorter clip is more beneficial. It is also possible that tailored videos that provide content that can be selected based on participants’ questions such as in Meropol et al.’s [20] study may be more effective than general videos. Additionally, it is possible that video is least accessible to those who stand to benefit most, if socioeconomic factors linked to literacy are also associated with more limited access to smartphone models, data plans, and/or availability of a computer in the home.

An unexpected, but notable finding came from the researcher experience which suggested that email contact prior to the recruitment phone call, regardless of group allocation, helped promote participants’ engagement with the study. This finding is contrary to the Cochrane Review by Mapstone et al. [30] who reported that pre-warning participants was not beneficial to enrolment, examining methods including sending a letter a week before [31], a postcard prior to sending a questionnaire [32] and leaving a voicemail [33]. However, none of these studies investigated the use of email to pre-warn participants, and our finding may be attributed to a shift in type of contact preference towards email. Specifically, as Samuels et al. [34] found that when asking subjects how they would like to be contacted for future research studies 58% opted for an email whereas only 16% opted for a phone call.

### Challenges, limitations and reflections on SWAT

We encountered a number of challenges in implementing the SWAT during an active RCT.

Whilst it is an advantage that the SWAT was responsive to recruitment challenges as they arose, the timeframe to implement the study significantly undermined the amount of data that could be collected. From initial idea to implementing the SWAT, the entire process took 13 months leaving just 4 months of data collection. This involved collaboration with the PPI group, submitting a substantial ethics amendment, creating the video clip and gaining local approvals from each of the trial sites. Thus, the sample size was small and this limited inferences that could be gleaned from the study. Future research would benefit from including SWAT procedure in the original ethics application and holding PPI consultations in the first instance to avoid delays. Indeed, in the UK, the NIHR Health Technology Assessment (HTA) programme now encourages the inclusion of SWAT protocols in funding proposals for host trials. To this end, the SWAT template provided by Smith et al. [35] may provide a helpful template that can be referenced as part of the host trial’s protocol. This would allow the opportunity to monitor the effectiveness of recruitment strategies from the start and for trials to determine the relative benefits of different strategies. However, there are also likely to be cases when SWATs will provide opportunities to test innovations that respond to unexpected challenges as they arise. Thus, efforts to increase the efficiency of ethical review and local approvals may be crucial to realising the benefit of SWATs.

The SWAT also added time into the recruitment process and an additional step, an email contact, that had not been completed previously. This had the effect of a cumulative increase in workloads in the context of demanding recruitment targets and deadlines. Combined with the randomisation allocation which changed weekly, this may have increased the likelihood of human error. This can be seen in the randomisation phase where six participants were contacted with the incorrect study allocation. Embedding the SWAT at the beginning of the trial could provide further time for training; alternatively, automating aspects of the recruitment process could also minimise the scope for human error.

It was difficult to control for a number of extraneous factors. For example, the number of contacts was conflated by the temporal factor of recruitment deadlines, such that the frequency of contacts increased in line with monthly deadlines. Ideally, the number of times the study team contacted participants would be standardised; however, this is not practical in the context of target-driven recruitment. Similarly, it was difficult to prioritise the interviews with participants about their SWAT experience at the risk of overburdening participants and without undermining the time allocated to outcome assessment. Thus, less interview data were collected than expected, particularly in the video condition, and this may have limited insight. Moreover, whilst participants were generally positive about the information they received through the SWAT, it would have been helpful to find out why they may not have watched the full duration of the video. Due to the limited sample size, we were unable to control for the initial contact type participants received during screening. For example, potential participants either completed screening in person with a member of the wider team or received questionnaires in the post. Whilst these were not notably different between groups, further research may benefit from controlling for extraneous factors of this nature. Moreover, participants were highly educated and often women; it is difficult to generalise these findings to other types of participants who may have different experiences which would influence their engagement with written material or video content.

The optimal design would be to test the video clip in isolation against the PIS to identify effects on recruitment. However, due to ethics requirements, participants had to receive the PIS prior to the baseline visit limiting our ability to formally test the video on recruitment. In a time of increased use of multimedia, it would be instructive to formally test whether there are complete alternatives to standard PIS that may be more acceptable and effective whilst being ethically robust. We were also unable to gain feedback from families that may have viewed the initial information but not enrolled in the study, which limits our understanding of participants’ reasons for not engaging with the study.

### Implications for recruitment practice

Although somewhat contrary to our expectations, it was encouraging that the PIS appeared to be effective for this sample. Moreover, participants and researchers perceived an initial email contact, regardless of whether it included the PIS or video, as being helpful. This suggests that initial contacts of this kind may be helpful in facilitating initial conversations about research as well as decision making about taking part. It would be helpful to test this formally using a SWAT design in future studies.

Future research may be focused on testing the effectiveness of a video clip as an initial recruitment contact rather than following a screening phase. Moreover, it would add value to evaluate this recruitment method in different demographic populations (e.g. those with lower literacy, children and young people, those with intellectual disabilities) in order to better ascertain utility. The inclusion of data regarding participants’ engagement with a PIS would also be helpful to identify potential areas for improvement and general levels of engagement.

We found involvement with our PPI group extremely helpful in developing the video and SWAT study. We strongly recommend that other researchers involve PPI groups when developing patient facing materials and designing trial procedures. The video may have benefited from several rounds of user testing to optimise its potential; however, due to timeframes and potential burden on the PPI group, this was not possible on this occasion.

Future research could consider whether videos may be particularly helpful for specific groups, better utilised in shorter (e.g. 30 s) formats addressing specific topics (e.g. randomisation), or as a supplementary material after participants have read the PIS. It would also be of interest to examine the impact of either formats on participant retention.

## Conclusions

The inclusion of a video clip explaining the trial did not result in an increase in recruitment to the host trial of a parenting intervention. Future research could consider a shorter video clip and explore its effectiveness in different populations alongside collaboration with a PPI group.

## Supplementary information

**Additional file 1: Appendix 1.** Interview schedule for participants. **Appendix 2.** Interview schedule for researchers.

## Data Availability

The de-identified data included in the paper will be available 12 months following publication and for 5 years after the date of publication. Data will be made available to researchers who provide a methodologically sound and hypothesis-driven proposal and have the required institutional approvals in place to achieve the aims in the approved proposal. To gain access to the data, proposals should be directed to Professor Paul Ramchandani for approval by the investigator group. Requestors will be asked to sign a data access agreement. The study protocol and consent form will also be made available.

## Abbreviations

HS,HS: Healthy Start, Happy Start
NIHR: National Institute for Health Research
NHS: National Health Service
PPI: Patient and public involvement
PIS: Participant information sheet
RCT: Randomised controlled trial
SWAT: Study within a trial
VIPP-SD: Video-feedback intervention to promote Positive Parenting and Sensitive Discipline
