# Supplementary material for: Does a video clip enhance recruitment into a parenting trial? Learnings from a study within a trial
Source: Trials. 2020 Oct 15;21:856. doi: 10.1186/s13063-020-04779-0 (PMC7558733; doi:10.1186/s13063-020-04779-0)
Supplement: Supplementary file 1 — Additional file 1: Appendix 1. Interview schedule for participants. Appendix 2. Interview schedule for researchers. [file 13063_2020_4779_MOESM1_ESM.docx]

**Appendix 1:** Interview schedule for participants

1. How helpful did you find the initial information about the study?
2. Did you watch the vide? (asked if people were in video group)
3. Did you find the initial information useful in understanding the research?
4. Did the initial information you received influence your decision to take part in the research? Why?
5. Is there anything else you would like to say or add?

**Appendix 2:** Interview schedule for researchers

1. Do you think the video was beneficial to participants? Why?
2. Do you think the video was time consuming to share with families?
3. How simple/difficult was sharing the video Clip?
4. How useful was the video to the consent process?
5. Anything you would change about the video or do differently in the future?
6. Do you have anything else you would like to add?
